# Hospital Outbreak of Carbapenem-Resistant *Klebsiella pneumoniae* Producing New Delhi Metallo-Beta-Lactamase — Denver, Colorado, 2012

**Published:** 2013-02-15

**Authors:** Larissa Pisney, Michelle Barron, Sarah Jackson Janelle, Wendy Bamberg, Duncan MacCannell, Alexander Kallen, Carolyn Gould, Brandi Limbago, Erin Epson, Joyanna Wendt

**Affiliations:** Div of Infectious Diseases, Univ of Colorado; Colorado Dept of Public Health and Environment; Antimicrobial Resistance and Characterization Laboratory; Div of Healthcare Quality Promotion, National Center for Emerging and Zoonotic Infectious Diseases; EIS officers, CDC

On August 16, 2012, the Colorado Department of Public Health and Environment was notified of two patients at an acute-care hospital in Denver with carbapenem-resistant Enterobacteriaceae (CRE), specifically *Klebsiella pneumoniae* (CRKP), isolated from respiratory specimens during July–August. Both isolates produced New Delhi metallo-beta-lactamase (NDM). A review of microbiology records identified a third patient with NDM-producing CRKP isolated from a respiratory specimen, admitted in May. Active surveillance cultures in September identified an additional five patients colonized with NDM-producing CRKP. An investigation was launched by the hospital and the Colorado Department of Public Health and Environment to guide infection control measures and limit transmission.

A case was defined as NDM-producing CRE isolated from clinical or active surveillance cultures collected from a patient while hospitalized during January 1–October 30, 2012. Medical records were reviewed for clinical and epidemiologic characteristics. Relatedness of isolates was evaluated by pulsed-field gel electrophoresis (PFGE).

The eight patients were aged 23–75 years and had been hospitalized at one or more of 11 different units in the hospital for a median of 18 days (range: 12–83 days) before CRKP identification. Three were treated for CRKP infection, and five were found to be asymptomatically colonized; none died. Initial isolates were resistant to all antimicrobials except tigecycline, to which all were susceptible. Colistin minimum inhibitory concentrations for six isolates were low (≤2 *μ*g/mL), suggesting this agent might be a treatment option. All isolates were highly related by PFGE. Epidemiologic tracing to determine temporal overlap of patients on units in the hospital indicated multiple transmission events had occurred, and three units were likely transmission sites. Acquisition of NDM-producing CRE by some patients was not explained by direct overlap and suggested that undetected, asymptomatically colonized patients were involved in some transmission routes. How NDM-producing CRE was introduced to the facility is unclear.

NDM, a carbapenemase enzyme first described in 2009 in a patient who had received medical care in India (1), has since been detected and reported worldwide (2). In the United States, before this outbreak, only 16 isolates in clusters with two or fewer cases had been identified since 2009; 14 isolates were from patients who had received medical care in endemic (South Asian) regions. The cases described here represent the largest U.S. outbreak of NDM-producing CRE to date, highlighting the risk for spread of these organisms among persons receiving medical care inside the United States. Evidence that undetected, asymptomatically colonized patients likely contributed to the size of the outbreak highlights the importance of timely active surveillance cultures when CRE is identified to direct infection control measures and limit further transmission (3).

## References

[1] Yong D, Toleman MA, Giske CG (2009). Characterization of a new metallo-beta-lactamase gene, *bla*_NDM-1_, and a novel erythromycin esterase gene carried on a unique genetic structure in *Klebsiella pneumoniae* sequence type 14 from India. Antimicrob Agents Chemother.

[2] Wilson ME, Chen LH (2012). NDM-1 and the role of travel in its dissemination. Curr Infect Dis Rep.

[3] CDC (2012). 2012 CRE toolkit: guidance for control of carbapenem-resistant Enterobacteriaceae (CRE).

